# Humoral and cellular immune response from first to fourth SARS-CoV-2 mRNA vaccination in anti-CD20-treated multiple sclerosis patients—a longitudinal cohort study

**DOI:** 10.3389/fimmu.2024.1432348

**Published:** 2024-09-05

**Authors:** Frederik Novak, Anna Christine Nilsson, Emil Birch Christensen, Caroline Louise Stougaard, Mike Bogetofte Barnkob, Dorte K. Holm, Agnes Hauschultz Witt, Keld-Erik Byg, Isik S. Johansen, Christian Nielsen, Tobias Sejbaek

**Affiliations:** ^1^ Department of Neurology, Hospital Southwest Jutland, University Hospital of Southern Denmark, Esbjerg, Denmark; ^2^ Department of Regional Health Research, University of Southern Denmark, Odense, Denmark; ^3^ Clinical Immunology Research Unit, Department of Clinical Immunology, Odense University Hospital, Odense, Denmark; ^4^ Department of Clinical Research, University of Southern Denmark, Odense, Denmark; ^5^ Centre for Cellular Immunotherapy of Haematological Cancer Odense (CITCO), Odense, Denmark; ^6^ Department of Neurology, Hospitalsenhed Midt, Viborg, Denmark; ^7^ Department of Rheumatology, Odense University Hospital, Odense, Denmark; ^8^ Department of Infectious Diseases, Odense University Hospital, Odense, Denmark

**Keywords:** multiple sclerosis, anti-CD20, mRNA vaccination vulnerable population, SARS-CoV-2, ocrelizumab concentration, breakthrough infection, humoral immune response, cellular immune activation

## Abstract

**Background:**

This study examines the humoral and cellular response in multiple sclerosis (MS) patients on anti-CD20 therapy before and after the 1st to 4th BNT162b2 mRNA SARS-CoV-2 vaccination and the relationship with breakthrough infection.

**Methods:**

Participants with McDonald 2017 MS that were treated with ocrelizumab were included. The study duration was throughout the COVID-19 pandemic until four months after fourth mRNA SARS-CoV-2 vaccination (BNT162b2). Longitudinal blood samples were analysed for: IgG antibodies of SARS-CoV-2 spike anti-receptor binding domain (anti-RBD), nucleocapsid IgG antibodies (anti-N) and activation induced marker expressing CD4+, CD8+ T-cells and concentration of ocrelizumab and anti-drug antibodies. Incidences of breakthrough infection were confirmed with SARS-CoV-2 PCR tests.

**Results:**

The rate of anti-RBD positive participants increased substantially between the third and fourth vaccination from 22.2% to 55.9% (median 54.7 BAU/mL; IQR: 14.5 – 221.2 BAU/mL and 607.7 BAU/mL; IQR: 29.4 – 784.6 BAU/mL, respectively). Within the same period 75% of participants experienced breakthrough infection. The fourth vaccination resulted in an additional increase in seropositive individuals (64.3%) (median 541.8 BAU/mL (IQR: 19.1-1007 BAU/mL). Breakthrough infection did not influence the cellular response without a significant change after the fourth vaccination. During the study period two participants had detectable anti-N, both after the fourth vaccination. No correlation was found between serum concentration of ocrelizumab and the humoral and cellular response.

**Discussion:**

Low levels or absence of specific anti-RBD following vaccination, with a significant increase after breakthrough infections and boosted by the fourth vaccination. T-cell reactivity remained sustained and unaffected by breakthrough infections.

## Introduction

The immunological response to SARS-CoV-2 mRNA vaccination has been extensively studied investigated in healthy individuals since the introduction of the vaccines early in 2021 (1). However, the memory response in patients with multiple sclerosis (MS) receiving B-cell-depleting therapy is less thoroughly characterized (2, 3). Since the emergence of SARS-CoV-2, it has evolved substantially. The alpha, delta, and omicron variants all mutated, resulting in an increasing number of amino acid variants in the Spike protein. This has led to a greater antigenic distance from the ancestral SARS-CoV-2 virus for which vaccines like BNT162b2, mRNA-1273, and Ad26.COV2.S were originally developed to provide protection (4–6). The ongoing evolution of the SARS-CoV-2 Spike domain has consequently led to higher antibody evasion and increased transmission rates despite high levels of adherence to vaccination programs (7, 8). However, breakthrough infections with the Omicron variants have not resulted in an increase in disease severity in patients treated with B-cell depleting anti-CD20 therapy, emphasizing the role of other cellular immune responses to SARS-CoV-2, notably T cells (9–11). Vaccines are generally still regarded as an effective prophylactic treatment against severe disease (12, 13), presumably due to a continued T-cell response across variants (14–16).

Research has demonstrated a significant impairment in the antibody response among MS patients undergoing anti-CD20 therapies compared with healthy controls, whereas the T-cell response remains comparable with that of healthy individuals (17, 18). With the emergence of the Omicron variants as the predominant strains in the pandemic, the incidence of breakthrough infections has become evident even among vaccinated individuals.

The levels of B cells and the duration since the last infusion of anti-CD20 antibodies directly influence the production of antibodies following vaccination. Serum concentrations of ocrelizumab have thus been proposed as a biomarker for B-cell levels and may serve as a surrogate marker for humoral response following vaccination (19).

This study is a longitudinal, multicenter cohort study that follow participants with MS receiving anti-CD20 therapy from their first to fourth vaccination, including rates of infection confirmed by polymerase chain reaction (PCR).

The study aimed to compare humoral and cellular immune responses after a fourth SARS-CoV-2 vaccination in relation to previous vaccinations. Secondly, we wanted to evaluate the influence of SARS-CoV-2 PCR-confirmed infections on the immune response.

## Method

### Study population and design

Participants above 18 years of age were included from two Danish clinics with confirmed MS (2017 McDonald Criteria) receiving ocrelizumab (anti-CD20) therapy before treatment with the first mRNA SARS-CoV-2 vaccination (BNT162b2). The participants were followed prospectively throughout the pandemic until they received the fourth vaccination booster. The results from the first to third vaccinations and clinical outcomes of breakthrough infection are previously published (9–11).

Similarly to the previous studies, no other immunosuppressive treatment beyond infusion and relapse-related methylprednisolone was given to the participants during this study. All participants followed standard clinical practice by their treating neurologist and the interval of time between the second, third, and fourth vaccinations was not standardized (4).

### Sample collection

**Figure 1:**
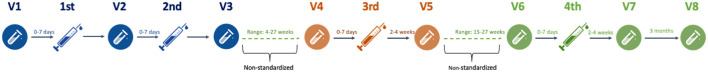
Study flowchart. Graphical illustration of the study flowchart from first to fourth SARS-CoV-2 mRNA vaccinations and blood samples before and after injections. V, visit number.

Blood samples were collected at eight time points (visit 1–8). The visit overview is depicted in **Figure 1**. SARS-CoV-2 IgG anti-spike receptor binding domain (RBD) (anti-RBD) and SARS-CoV-2 nucleocapsid IgG antibody (anti-N) responses were measured at each visit. Anti-RBD levels from 0 to 7 days before the first vaccination (V1) were compared with the levels 0–7 days before the second booster vaccination (V2) and 2–4 weeks after the second booster vaccination (V3) concluding the first vaccination cycle at the beginning of the pandemic (10). Likewise, the levels 0–7 days before the third booster vaccination (V4) were compared with the levels 2–4 weeks after the third booster (V5) (9). Finally, the levels 0–7 days before the fourth booster vaccination (V6) were compared with 2–4 weeks (V7) and again 3 months (V8) after the fourth booster vaccination. Serum concentrations of ocrelizumab and antidrug antibodies (ADA) were analyzed at visits 1, 2, and 4.

Peripheral blood mononuclear cells (PBMCs) were collected at V1, V3, V5, V7, and V8. Equally, activation-induced marker expressing CD4+ and CD8+ T-cells were compared between each visit.

### Data collection

Blood samples were collected following international guidelines for biobanking. Venous blood was procured from a cubital vein into evacuated K2-EDTA or heparinized tubes. Plasma was aliquoted in 500-μL Sarstedt polypropylene tubes and stored at −80°C until batch analysis (10, 20).

### Human peripheral blood mononuclear cell isolation

PBMCs were isolated from whole blood using density gradient centrifugation (Lymphoprep™) and cryopreserved using a DMSO-containing freezing medium and stored at −196°C until flow cytometric analysis (9).

### Antibody assay and flow cytometry

We measured IgG antibodies against the SARS-CoV-2 spike RBD in plasma samples with the SARS-CoV-2 IgG II Quant assay (Abbott Laboratories), which is a quantitative chemiluminescent microparticle immunoassay (21). The assay was performed using the Abbott Alinity I platform according to the manufacturer’s instructions. This assay has shown excellent correlation with the first WHO (World Health Organization) International Standard for anti-SARS-CoV-2 immunoglobulin (NIBSC code 20/136) (22), enabling the issuing of immunogenicity results in standardized units; binding antibody units (BAU)/mL for a binding assay format as the SARS-CoV-2 IgG II Quant assay. The mathematical relationship of the Abbott AU/mL unit to the WHO BAU/mL unit follows the equation BAU/mL = 0.142 × AU/mL, corresponding to a lower limit of detection cutoff at 7.1 BAU/mL. The range of measurement with the described assay is 7.1 BAU/mL–5,680 BAU/mL.

Anti-N were determined using the SARS-CoV-2 IgG assay, a qualitative chemiluminescent microparticle immunoassay (Abbott Laboratories), to detect patients who were convalescent after COVID-19. Samples were tested according to the manufacturer’s instructions using a fully automated system. Samples ≥1.4 were interpreted as positive for anti-N.

### PCR testing and breakthrough infection

Results of the SARS-CoV-2 PCR test were collected throughout the study. Data were collected from the Danish National Microbiology Database MiBa (Statens Serum Institut, Copenhagen, Denmark), as described in the study reporting breakthrough infection and clinical outcomes (11).

### Serum concentration and antidrug antibody assay

The drug concentrations of ocrelizumab (ng/mL) were measured by quantitative assay. Qualitative assay determined ADAs with sandwich-ELISA. Samples were confirmed positive for antibodies to ocrelizumab if the percentage decrease in signal is at or above 38% using confirmatory reagent (Supplementary 1). Ocrelizumab concentration and ADA analyses were performed at PPD laboratories, Richmond, Virginia, US.

### T-cell assay

PBMCs were thawed and resuspended in RPMI 1640 basal medium with 10 UI/mL DNase I. The cells were hereafter washed resuspended and equilibrated in RPMI 1640 basal medium with 10% FBS and 1% penicillin/streptomycin containing 1:1,000 brefeldin A. PBMCs were plated at 1 × 10^6^ cells/well in 96-well round-bottom plates and stimulated for 4 h with 1 µg/mL spike peptide pools (Miltenyi Biotec, catalog 130-126-701). In all assays, 2% DMSO vehicle control was used for no stimulation and anti-CD3/anti-CD28 beads (1:100) as positive control.

Following stimulation, cells were washed in PBS with 1% BSA and stained for 30 min at 4°C in the dark with the following antibody panel: CD3 APC-Fire750 (1:600) CD4 SparkBlue-550 (1:400), CD8 BV570 (1:200), CD14 Pacific Blue (1:200), CD19 Pacific Blue (1:200), CCR7 BUV737 (1:50), CD45RA BUV395 (1:600), and CD69 BV650 (1:100), all BD Biosciences.

Cells were then washed in PBS with 1% BSA, fixed, and permeabilized using 1× Fix/Perm solution (BD) and 1× Perm buffer (BD) before staining with intracellular cytokine antibodies against IFNy (PE; 1:50) and TNFα (APC; 1:50; both BD). Activation-induced marker (AIM) positive T cells were defined by the expression of IFNy or TNFα of activated (CD69 positive) non-naïve (CD45RA−CCR7+, CD45RA−CCR7− or CD45RA+CCR7−) T cells (CD3+CD4+ or CD3+CD8+). AIM expression following spike peptide stimulation was subtracted from AIM expression following stimulation with no peptide-negative control to yield the frequencies of spike-specific T cells.

### Standard protocol approvals, registrations, patient consents, and monitoring

Written and oral consent was given from all participants. The study followed national laws adhering to good clinical practice and was approved by the Danish National Committee on Health Research Ethics (Protocol no. S-20200068C) and Danish Data Protection Agency (journal no. 20/19,878) (9, 10).

### Statistical analysis

All data were analyzed for normal distribution with D’Agostino–Pearson test. Continuous data were presented as the median with interquartile range (IQR). Then, anti-RBD values were converted with log-transformation to account for skewness of data, since a large proportion of the results were below the cutoff <7.1 BAU/mL. Mixed-effect analysis test was used to compare the results between visits. Simple linear regression analysis was used to determine the correlation between anti-RBD and variables such as ocrelizumab serum concentrations and the humoral response. The level of significance was defined as p<0.05.

## Results

### Study cohort

The median patient age in this cohort of MS patients was 49 years (range: 27–68 years). Participant baseline demographic and clinical characteristics are shown in **Table 1**. A total of 41 participants participated and were analyzed for IgG antibodies against SARS-CoV-2 in relation to all four mRNA vaccinations. CD4+ and CD8+ spike-specific T cells were analyzed from 26 (63.4%) participants. Blood samples were collected from 23/February/2021, to 16/September/2022, a period in which the Delta and Omicron variants dominated in Denmark. All participants were vaccinated with BNT162b2.

**Table 1:** Baseline clinical and demographic characteristics.

No. of participants	41
Female, n (%)	29 (70.7)
Median age, years (IQR)	49 (27–68)
Time interval between 4th vaccine and following ocrelizumab infusion, median weeks (IQR)	10.9 (3.6–14.4)
Time interval between 3rd and 4th vaccines, median weeks (IQR)	24.9 (20.0–26.3)
Time interval between breakthrough infection and 4th vaccine, median weeks (IQR)	8.5 (3.68–12.04)
Time interval between visits, median weeks (IQR)	
V2–V3	5.0 (4.8–5.5)
V3–V4	21.1 (14.5–23.0)
V4–V5	4.1 (3.9–4.7)
V5–V6	21.0 (18.8–22.2)
V6–V7	4.0 (3.4–5)
V7–V8	9.2 (9.0–11.5)

### Antibody levels in relation to the first three vaccinations

**Figure 2:**
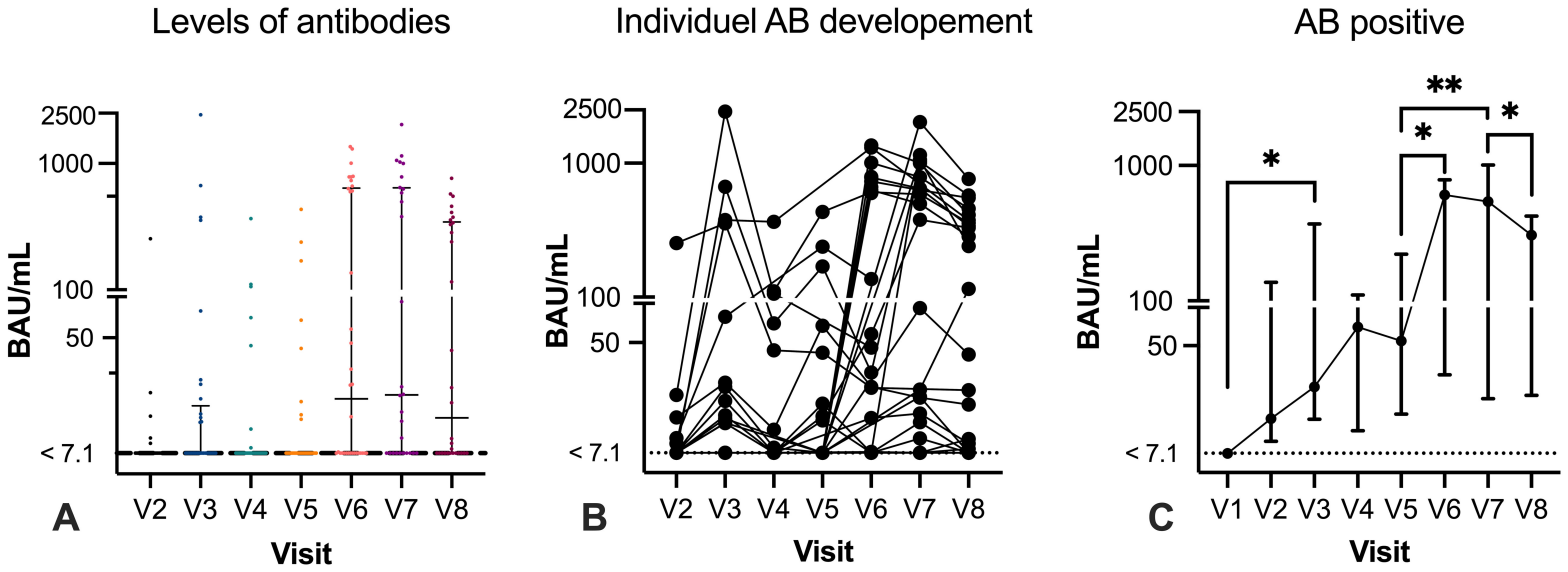
Humoral response with specific antibody levels before and after vaccination. Specific SARS-CoV-2 IgG anti-RBD levels are given in binding antibody level (BAU)/mL (y-axis, log 10). Graph **(A)** illustrates SARS-CoV-2 IgG anti-RBD levels at each visit. The lowest level of detection was 7.1 BAU/mL and is indicated with a dotted line. Results <7.1 are indicated on the line. Graph **(B)** illustrates antibody levels in paired samples. Graph **(C)** demonstrates the median and interquartile range (IQR) of all samples. Mixed-effect analysis test was used to compare the results between visits. Only significant results are depicted in graph **(C)**. *p < 0.05; **p < 0.01.

None of the participants had a previous history of COVID-19 prior to study inclusion, had no prior positive qPCR test, and were anti-N and anti-RBD negative at V1 (baseline). After the first mRNA vaccination, at V2, 12.8% of participants seroconverted and had developed anti-RBD with a median of 13.3 BAU/mL (IQR: 8.8 BAU/mL–136.9 BAU/mL), which increased to 33.3% after the second vaccination. At V3, the median anti-RBD level was 23.7 BAU/mL (IQR: 13.2 BAU/mL–370.7 BAU/mL). From the follow-up visit of the second vaccination to before the third vaccination (V3 to V4), the median time was 21.1 weeks (IQR: 14.5–23.0 weeks). At V4, SARS-CoV-2 antibody titers were increased compared with V3 in seropositive participants (median: 70.2 BAU/mL; IQR: 10.7 BAU/mL–110.4 BAU/mL), but the frequency of seropositive patients decreased to 20% of participants. After the third vaccination (V5), 22.2% of participants were seropositive with a median of 54.7 BAU/mL (IQR: 14.5–221.2 BAU/mL). The SARS-CoV-2 antibody titers increased significant from V1 to V3 (p=0.0324) (**Figure 2**) and no statistical difference was found from V3 to V5.

### Antibody levels before the fourth vaccination

The proportion of participants with detectable anti-RBD at V6 (n=34) increased substantially before the fourth booster vaccination, where 55.9% of participants had measurable anti-RBD. Of the seropositive participants, the median antibody level was 607.7 BAU/mL (IQR: 29.4 BAU/mL–784.6 BAU/mL). Compared with V5, anti-RBD were increased, and nine participants seroconverted to a detectable anti-RBD (784.4 BAU/mL; IQR: 647.4–898.9) and two participants converted from a positive to negative response (**Figure 2**). There was a significant increase in the SARS-CoV-2 antibody response from V5 to V6 (p=0.0148). The median time between third and fourth vaccination was 24.9 weeks (IQR: 20 – 26.3) (**Table 1**).

### Antibody levels after the fourth vaccination

After the fourth SARS-CoV-2 vaccination (V7) (median 4 weeks; IQR: 3.6–5), 18 out of 28 (64.3%) participants demonstrated a seropositive response with a median of 541.8 BAU/mL (IQR: 19.1–1007 BAU/mL) (**Figure 2C**). Comparing the results after the third and fourth vaccinations (V5 and V7), paired results were available in 25 participants, of whom 5 out of 25 (20%) had detectable anti-RBD at both V5 and V7 with a median of 378.9 BAU/mL (range: 14.01 BAU/mL–806.3 BAU/mL) after the fourth vaccination. However, a total of 11 previous seronegative participants at V5 developed a detectable anti-RBD response after the fourth vaccination (median 630.3 BAU/mL; IQR: 21.8–1001). At V8 (n=30), detectable anti-RBD was measured in 18 participants (60%) but the level decreased to a median of 306.6 BAU/mL (IQR: 20.3 BAU/mL–422.3 BAU/mL) after a median of 9.2 weeks (IQR: 9.0–11.5 weeks). Between the third and fourth vaccinations (V5 to V7), the SARS-CoV-2 antibody titers significantly increased (p=0.0032) before decreasing between V7 and V8 (p=0.0113). The time between the fourth vaccination (R^2^= 0.008, p=0.660) or the nearest anti-CD20 infusion (R^2^ = 0.010, p=0.709) did not affect the SARS-CoV-2 antibody titers (**Figure 2**).

### Breakthrough infection and the relation to the fourth vaccination

PCR test results were available from 36 participants. The breakthrough infection rate was 75% where two participants had a positive PCR test after they received the fourth vaccination (2 and 5 weeks, respectively). Analysis of anti-N only found reactivity in two participants, one at V7 and one at V9. The remaining 25 participants had positive test results before the fourth vaccination with a median of 8.5 weeks (IQR: 3.68–12.04 weeks). Of all tested participants, 12 (33.3%) infected participants had detectable anti-RBD at V6 (median 697.2; IQR: 600.9 BAU/mL–789.8 BAU/mL).

Infected participants with a seropositive response at V7 increased to 15 (41.7%), but SARS-CoV-2 antibody titers decreased in the same period with a median of 496.7 (IQR: 19.21 BAU/mL–784.9 BAU/mL). Similarly, SARS-CoV-2 antibody titers continued to decrease at V8 in the infected group of participants, where 14 participants had a median of 306.6 BAU/mL (34.53 BAU/mL–422.3 BAU/mL).

### Spike-specific CD4+ and CD8+ T cells

**Figure 3:**
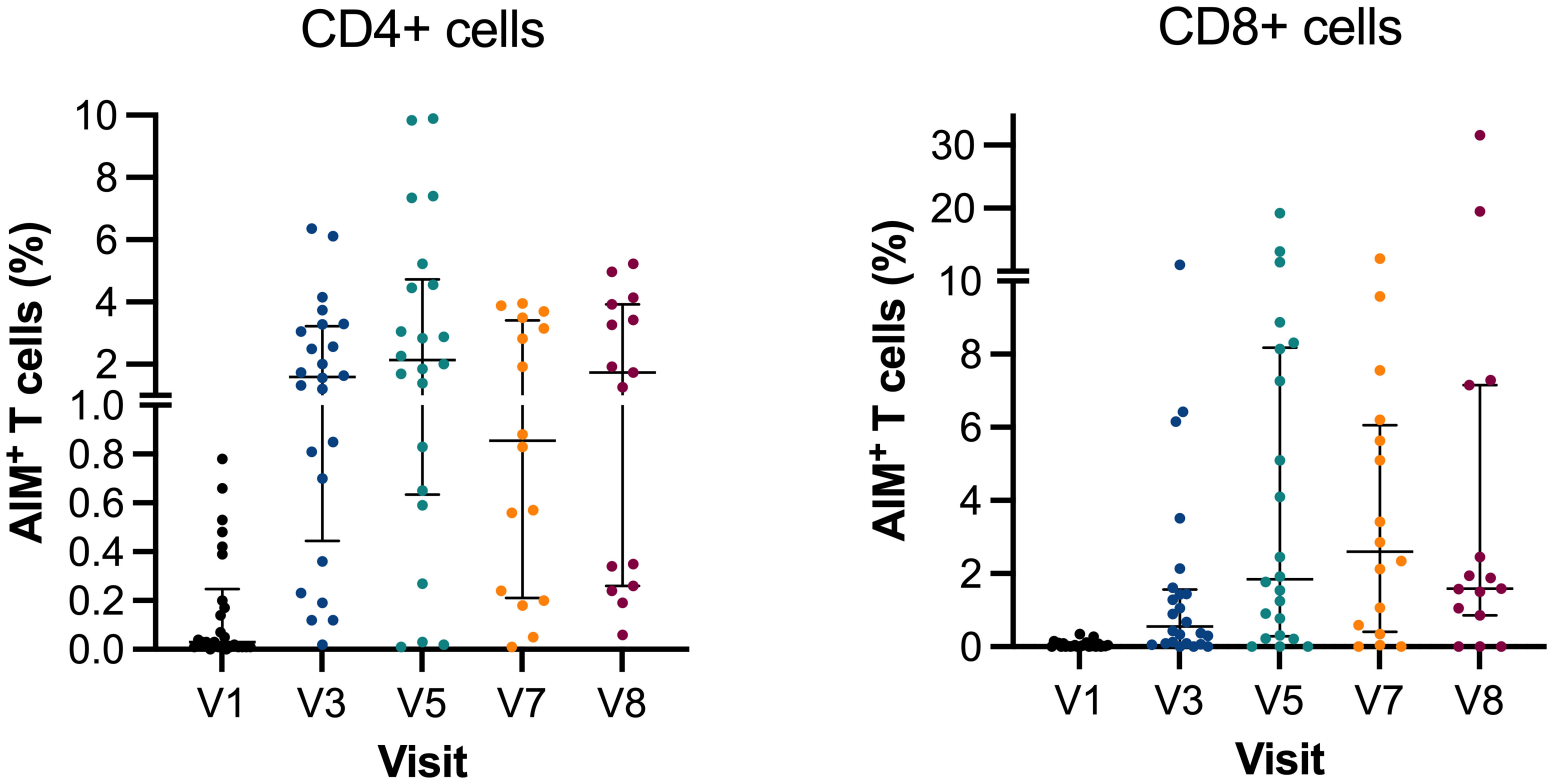
Activation-induced markers of CD4^+^ and CD8^+^ T cells. Scatter plot graph depicting frequencies of spike-reactive CD4^+^ and CD8^+^ cell median with IQR at V1, V3, V5, V7, and V8. Y-axis in two segments ranging 0%–1% and 1%–10% for CD4^+^ and 0%–10% and 10%–30% for CD8^+^. AIM, activation-induced markers of positive T cells.

The frequency of spike-specific CD4+ and CD8+ T cells at V1 (n=26) had a median of 0.03 × 10^9^ cells/L (IQR: 0.01–0.25) and 0.02 × 10^9^ cells/L (IQR: 0.01–0.08), respectively. As previously reported, the frequency increased rapidly at V3 (n=24), where the median of CD4+ was 1.6 × 10^9^ cells/L (IQR: 0.45–3.23) and 0.55 × 10^9^ cells/L (IQR: 0.09–1.57) for CD8+ spike-specific T cells (p ≤ 0.0001). The frequency continued to increase after the third vaccination booster, and results at V5 (n=22) demonstrated a CD4+ median of 2.14 × 10^9^ cells/L (IQR: 0.64–4.73) and CD8+ median of 1.85 × 10^9^ cells/L (IQR: 0.29–8.18). The increase was only significant for CD8+ cells compared with V3 (p=0.0489) (**Figure 3**).

When comparing the spike-specific T cells continuously after the third and fourth vaccinations, the frequency decreased but not between V5 and V7 (n=16), where the median of CD4+ and CD8+ was 0.86 × 10^9^ cells/L (IQR: 0.21–3.42) and 2.61 × 10^9^ cells/L (IQR: 0.41–6.06), respectively. Similarly, there were changes in spike-specific T cells at V8 (n=15) (median 1.73 × 10^9^ cells/L; IQR: 0.26–3.93 and 1.59 × 10^9^ cells/L; IQR: 0.86–7.16). Breakthrough infection did not affect frequencies of spike-specific T cells, and no difference was found between V5, V7, and V8. At V7, the time between the third and fourth vaccinations did not correlate with the T-cell levels (CD4+: R^2^= 0.01, p=0.700; CD8+: R^2^ = 0.0001, p=0.969). Likewise, the timing between vaccination and infusion did not affect the T-cell response (CD4+: R^2^= 0.0425, p=0.444; CD8+: R^2^ = 0.0004, p=0.940).

### Serum concentrations of ocrelizumab (PK levels)

The serum was analyzed at V1, V2, and V4 before the first three mRNA vaccinations. The median concentration of ocrelizumab at V1 was 3,870 ng/mL (IQR: 1,630–5,915). The serum concentration decreased at V2 with a median of 1,630 ng/mL (IQR: 683.5–2,830) (p ≤ 0.0001), and lastly, an increase at V4 with a median of 3,870 ng/mL (IQR: 1,710–19,500) (p=0.0003). There was no correlation between serum concentrations of ocrelizumab and the subsequent levels of SARS-CoV-2 antibody titers and frequency of spike-specific CD4+ and CD8+ T-cells at V2 (R^2^ = 0.03, p=0.416), V3 (R^2^ = 0.41, p=0.529), and V5 (R^2^ = 0.02, p=0.575). ADA was not detected at any timepoint in the participants during the study.

## Discussion

This longitudinal cohort study examines the humoral and cellular immune response pre- and post-administration of four doses of SARS-CoV-2 mRNA vaccines. A subset of participants (75%) experienced clinical and PCR-confirmed infection between the third and fourth vaccinations. The findings indicate that the individuals treated with ocrelizumab displayed lower proportions and levels of SARS-CoV-2-specific antibodies compared with those in healthy controls. Notably, anti-RBD levels significantly increased following clinical and PCR-confirmed infection, aligning with observations in healthy controls (23–25). T-cell reactivity post-vaccination was similar to that of healthy controls and remained unaffected by clinical infection.

Initially, the study assessed longitudinal levels of anti-RBD and observed a remarkable increase in both the development of levels and seropositivity-specific anti-RBD before and after the fourth vaccination. Following the fourth vaccination, over half (55.9%) of the participants had specific anti-RBD, in contrast to 22.2% after the third vaccination. Moreover, the median levels of anti-RBD increased from 54.7 BAU/mL to 541.8 BAU/mL post-infection and fourth vaccination. Our findings suggest that breakthrough infections have a greater impact on the humoral response more profoundly than continuous vaccination. Additionally, patients experiencing breakthrough infection did not elicit anti-N antibodies (except in two cases), indicating that the significant boost in anti-RBD after infection could be a result of previous vaccinations. These findings underline previous research indicating that initial infection boosts the humoral response to subsequent vaccination, even in patients undergoing treatment with ocrelizumab (23–25) (**Figure 2**). Furthermore, novel bivalent vaccines have demonstrated greater efficacy in preventing breakthrough infections caused by current virus variants in immunosuppressed individuals (26). However, breakthrough infections did not lead to severe disease progression in participants with B-cell-depleted participants (11). Evaluating the seroconversion for detectable humoral responses, the most significant change occurred between V5 and V6 (median 21 weeks), with the proportion increasing from 22.2% to 55.9%, respectively (**Figure 2**). Among the participants with detectable anti-RBD at V6, prior to the fourth mRNA vaccination, 77.8% had been seronegative after the third vaccination, implying that the alteration in the humoral response was not influenced by vaccination. Consequently, the seroconversion was attributable to the occurrence of SARS-CoV-2 infection. Following administration of the fourth vaccination at V7, the number of seropositive participants peaked with 64.3% displaying detectable anti-RBD, indicating the ongoing efficacy of the vaccine in inducing seroconversion in participants receiving anti-CD20 therapy. The median interval between V7 and V8 was 9.2 weeks, during which three participants transitioned to a seronegative status. These individuals had low antibody titer <20 BAU/mL at V7, confirming the trend of decreasing levels of circulating anti-RBD post-vaccination, observed in both immunosuppressed and healthy individuals (9, 27, 28).

Anti-N was analyzed because of the potential utility as a marker in the screening and diagnosis of patients with COVID-19. Only two participants exhibited anti-N at two timepoints. This prevalence is notably lower compared with findings from involving healthy controls and solid organ transplant recipients. Unfortunately, anti-N was not assessed at the time of infection (range: 0.3–16.4 weeks). However, the detection in immunosuppressed patients up to 9 months postinfection suggests limited or negligible clinical significance in B-cell-depleted patients (29, 30).

We observed a persistent T-cell response following the administration of the second to fourth doses of SARS-CoV-2 vaccine compared with the earlier visits. The T-cell reactivity appeared to be better preserved than antibody levels among the study participants. The findings align with those reported in studies focusing on the initial three vaccinations in both immunosuppressed and healthy individuals (9, 11, 31, 32). **Figure 3** illustrates that the cellular response plateaued after the initial vaccination and has remained stable through V5–V8, regardless of the following vaccinations and breakthrough infection.

There was no correlation between anti-RBD or the frequency of spike-specific T-cell response and the duration between the third and fourth vaccinations. Similarly, the timing between vaccination and anti-CD20 infusion did not influence the immune response to vaccination. However, participants in our study received a standard interval of 24 weeks between ocrelizumab infusions, and the relationship between the time since the last infusion and antibody levels has primarily been observed in studies investigating extending dosing intervals (33, 34). This observation indicates that timing has minimal influence, contrary previous conclusions, highlighting instead the significance of breakthrough infections on the humoral and cellular reactivity observed in our participants (25). Serum concentrations of ocrelizumab were analyzed in 25 participants (61%) at V1, V2, and V4, corresponding to the periods before the first, second, and third vaccinations, respectively.

Finally, we performed a simple linear regression analysis of anti-RBD response and serum concentrations of ocrelizumab at all timepoints, revealing no significant correlation. This contrasts with a previous study that reported a significant association between antibodies and drug concentrations (19). However, the study reported results in arbitrary units (AU/mL), whereas our study utilized international standardized binding antibody units (BAU)/mL. Additionally, different assays were used in the two studies, which could contribute to the differences observed. In a recent study on extended interval dosing with ocrelizumab, the elimination of ocrelizumab in serum exceeds the infusion interval and absence of ocrelizumab in the blood of treated patients only occurs if the dosing interval is extended. B-cell repopulation almost only occurs if the dosing interval is extended. Thus, it limits the possibility of B-cell repopulation which is crucial for the humoral response within the current study design (35).

This study had certain limitations. The limited timeframe for collecting blood samples led to instances of missing samples. Furthermore, the T-cell analysis was conducted solely at one of the three MS centers, involving 26 participants. Additionally, the study did not include a healthy control group. In general, the number of participants is limited; however, the longitudinal design in the context of the pandemic is novel to current literature.

In conclusion, this longitudinal cohort study provides data on the humoral and cellular response from first to fourth mRNA SARS-CoV-2 vaccination. Overall, we observed low levels, or the absence of specific anti-RBD following vaccination, with a significant increase after breakthrough infections and the fourth vaccination. The T-cell reactivity remained sustained and unaffected by breakthrough infections. Furthermore, we observed no correlation between ocrelizumab concentration, and the levels of specific anti-RBD.

## Data Availability

The raw data supporting the conclusions of this article will be made available by the authors, without undue reservation.

## References

[1] Baden LR El Sahly HM Essink B Kotloff K Frey S Novak R. Efficacy and safety of the mRNA-1273 SARS-coV-2 vaccine. New Engl J Med. (2021) 384:403–16. doi: 10.1056/NEJMoa2035389 PMC7787219 33378609

[2] Disanto G Galante A Cantu M Sacco R Mele F Eisler JJ. Longitudinal postvaccine SARS-coV-2 immunoglobulin G titers, memory B-cell responses, and risk of COVID-19 in multiple sclerosis over 1 year. Neurology(R) neuroimmunology Neuroinflamm. (2023) 10:7–10. doi: 10.1212/NXI.0000000000200043 PMC9747147 36396447

[3] Sette A Crotty S. Immunological memory to SARS-CoV-2 infection and COVID-19 vaccines. Immunol Rev. (2022) 310:27–46. doi: 10.1111/imr.13089 35733376 PMC9349657

[4] Polack FP Thomas SJ Kitchin N Absalon J Gurtman A Lockhart S. Safety and efficacy of the BNT162b2 mRNA covid-19 vaccine. New Engl J Med. (2020) 383:2603–15. doi: 10.1056/NEJMoa2034577 PMC7745181 33301246

[5] Baden LR El Sahly HM Essink B Kotloff K Frey S Novak R. Efficacy and safety of the mRNA-1273 SARS-coV-2 vaccine. New Engl J Med. (2020) 384:403–16. doi: 10.1056/NEJMoa2035389 PMC7787219 33378609

[6] Sadoff J Gray G Vandebosch A Cárdenas V Shukarev G Grinsztejn B. Safety and efficacy of single-dose ad26.COV2.S vaccine against covid-19. New Engl J Med. (2021) 384:2187–201. doi: 10.1056/NEJMoa2101544 PMC8220996 33882225

[7] Li J Lai S Gao GF Shi W. The emergence, genomic diversity and global spread of SARS-CoV-2. Nature. (2021) 600:408–18. doi: 10.1038/s41586-021-04188-6 34880490

[8] Andrews N Stowe J Kirsebom F Toffa S Rickeard T Gallagher E. Covid-19 vaccine effectiveness against the omicron (B.1.1.529) variant. New Engl J Med. (2022) 386:1532–46. doi: 10.1056/NEJMoa2119451 PMC8908811 35249272

[9] Bajwa HM Novak F Nilsson AC Nielsen C Holm DK Østergaard K. Persistently reduced humoral and sustained cellular immune response from first to third SARS-CoV-2 mRNA vaccination in anti-CD20-treated multiple sclerosis patients. Multiple sclerosis related Disord. (2022) 60:103729. doi: 10.1016/j.msard.2022.103729 PMC8898195 35334278

[10] Novak F Nilsson AC Nielsen C Holm DK Østergaard K Bystrup A. Humoral immune response following SARS-CoV-2 mRNA vaccination concomitant to anti-CD20 therapy in multiple sclerosis. Multiple sclerosis related Disord. (2021) 56:103251. doi: 10.1016/j.msard.2021.103251 PMC8426319 34571415

[11] Novak F Bajwa HM Coia JE Nilsson AC Nielsen C Holm DK. Low protection from breakthrough SARS-CoV-2 infection and mild disease course in ocrelizumab-treated patients with multiple sclerosis after three mRNA vaccine doses. J Neurology Neurosurg Psychiatry. (2023) 94:934–7. doi: 10.1136/jnnp-2022-330757 PMC10579504 37185261

[12] Martinelli S Pascucci D Laurenti P. Humoral response after a fourth dose of SARS-CoV-2 vaccine in immunocompromised patients. Results of a systematic review. Front Public Health. (2023) 11:1108546. doi: 10.3389/fpubh.2023.1108546 37033069 PMC10076800

[13] Bar-On YM Goldberg Y Mandel M Bodenheimer O Amir O Freedman L. Protection by a fourth dose of BNT162b2 against omicron in Israel. New Engl J Med. (2022) 386:1712–20. doi: 10.1056/NEJMoa2201570 PMC9006780 35381126

[14] Nesamari R Omondi MA Baguma R Höft MA Ngomti A Nkayi AA. Post-pandemic memory T cell response to SARS-CoV-2 is durable, broadly targeted, and cross-reactive to the hypermutated BA.2.86 variant. Cell Host Microbe. (2024) 32:162–9.e3. doi: 10.1016/j.chom.2023.12.003 38211583 PMC10901529

[15] Skelly DT Harding AC Gilbert-Jaramillo J Knight ML Longet S Brown A. Two doses of SARS-CoV-2 vaccination induce robust immune responses to emerging SARS-CoV-2 variants of concern. Nat Commun. (2021) 12:5061. doi: 10.1038/s41467-021-25167-5 34404775 PMC8371089

[16] Sabatino JJ Jr. Mittl K Rowles WM McPolin K Rajan JV Laurie MT. Multiple sclerosis therapies differentially affect SARS-CoV-2 vaccine-induced antibody and T cell immunity and function. JCI Insight. (2022) 7:6–9. doi: 10.1172/jci.insight.156978 PMC8876469 35030101

[17] Wu X Wang L Shen L Tang K. Response of COVID-19 vaccination in multiple sclerosis patients following disease-modifying therapies: A meta-analysis. EBioMedicine. (2022) 81:104102. doi: 10.1016/j.ebiom.2022.104102 35759920 PMC9230320

[18] Mu Y Wu H Jiang Z Liu K Xue X Zhang W. Serological responses after a fourth dose of SARS-coV-2 vaccine in solid organ transplant recipients: A systematic review and meta-analysis. Vaccines. (2023) 11:1130. doi: 10.3390/vaccines11071130 37514946 PMC10385971

[19] van Kempen ZLE Hogenboom L Toorop AA Steenhuis M Stalman EW Kummer LYL. Ocrelizumab concentration is a good predictor of SARS-coV-2 vaccination response in patients with multiple sclerosis. Ann neurology. (2023) 93:103–8. doi: 10.1002/ana.26534 PMC9874752 36250739

[20] Teunissen CE Petzold A Bennett JL Berven FS Brundin L Comabella M. A consensus protocol for the standardization of cerebrospinal fluid collection and biobanking. Neurology. (2009) 73:1914–22. doi: 10.1212/WNL.0b013e3181c47cc2 PMC2839806 19949037

[21] Abbott. SARS-CoV-2 IgG II Quant for the use with Alinity i, Vol. 2021. (2021).

[22] Kumar A Bernasconi V Manak M de Almeida Aranha AP Kristiansen PA. The CEPI centralised laboratory network: supporting COVID-19 vaccine development. Lancet (London England). (2021) 397:2148–9. doi: 10.1016/S0140-6736(21)00982-X PMC8175055 34090600

[23] Andreano E Paciello I Piccini G Manganaro N Pileri P Hyseni I. Hybrid immunity improves B cells and antibodies against SARS-CoV-2 variants. Nature. (2021) 600:530–5. doi: 10.1038/s41586-021-04117-7 PMC8674140 34670266

[24] Goldberg Y Mandel M Bar-On YM Bodenheimer O Freedman LS Ash N. Protection and waning of natural and hybrid immunity to SARS-coV-2. New Engl J Med. (2022) 386:2201–12. doi: 10.1056/NEJMoa2118946 PMC9165562 35613036

[25] Verheul MK Vos M de Rond L De Zeeuw-Brouwer ML Nijhof KH Smit D. Contribution of SARS-CoV-2 infection preceding COVID-19 mRNA vaccination to generation of cellular and humoral immune responses in children. Front Immunol. (2023) 14:1327875. doi: 10.3389/fimmu.2023.1327875 38193077 PMC10773747

[26] Christophorou E Nilsson AC Petersen I Lindvig SO Davidsen JR Abazi R. Humoral antibody response following mRNA vaccines against SARS-CoV-2 in solid organ transplant recipients; a status after a fifth and bivalent vaccine dose. Front Immunol. (2023) 14:1270814. doi: 10.3389/fimmu.2023.1270814 38090591 PMC10711048

[27] Levin EG Lustig Y Cohen C Fluss R Indenbaum V Amit S. Waning immune humoral response to BNT162b2 covid-19 vaccine over 6 months. New Engl J Med. (2021) 385:e84. doi: 10.1056/NEJMoa2114583 34614326 PMC8522797

[28] Assawakosri S Kanokudom S Suntronwong N Chansaenroj J Auphimai C Nilyanimit P. Immunogenicity and durability against Omicron BA.1, BA.2 and BA.4/5 variants at 3-4 months after a heterologous COVID-19 booster vaccine in healthy adults with a two-doses CoronaVac vaccination. Heliyon. (2024) 10:e23892. doi: 10.1016/j.heliyon.2023.e23892 38226248 PMC10788509

[29] Gaeta A Angeloni A Napoli A Pucci B Cinti L Roberto P. Anti-N SARS-CoV-2 assays for evaluation of natural viral infection. J Immunol Methods. (2023) 518:113486. doi: 10.1016/j.jim.2023.113486 37156408 PMC10163944

[30] Søfteland JM Gisslén M Liljeqvist J Friman V de Coursey E Karason K. Longevity of anti-spike and anti-nucleocapsid antibodies after COVID-19 in solid organ transplant recipients compared to immunocompetent controls. Am J Transplant. (2022) 22:1245–52. doi: 10.1111/ajt.16909 PMC9906230 34860447

[31] Habek M Željko C Savić Mlakar A Bendelja K Rogić D Adamec I. Humoral and cellular immunity in convalescent and vaccinated COVID-19 people with multiple sclerosis: Effects of disease modifying therapies. Multiple sclerosis related Disord. (2022) 59:103682. doi: 10.1016/j.msard.2022.103682 PMC8824161 35158189

[32] Apostolidis SA Kakara M Painter MM Goel RR Mathew D Lenzi K. Cellular and humoral immune responses following SARS-CoV-2 mRNA vaccination in patients with multiple sclerosis on anti-CD20 therapy. Nat Med. (2021) 27:1990–2001. doi: 10.1038/s41591-021-01507-2 34522051 PMC8604727

[33] Disanto G Sacco R Bernasconi E Martinetti G Keller F Gobbi C. Association of disease-modifying treatment and anti-CD20 infusion timing with humoral response to 2 SARS-coV-2 vaccines in patients with multiple sclerosis. JAMA Neurology. (2021) 78:1529–31. doi: 10.1001/jamaneurol.2021.3609 PMC8461551 34554185

[34] Ali A Dwyer D Wu Q Wang Q Dowling CA Fox DA. Characterization of humoral response to COVID mRNA vaccines in multiple sclerosis patients on disease modifying therapies. Vaccine. (2021) 39:6111–6. doi: 10.1016/j.vaccine.2021.08.078 PMC8411370 34483021

[35] Novak F Bajwa HM Østergaard K Berg JM Madsen JS Olsen DA. Extended interval dosing with ocrelizumab in multiple sclerosis. Multiple Sclerosis J. (2024) 30:847–56. doi: 10.1177/13524585241245296 38646949

