# Supplementary material for: Humoral and cellular immune response from first to fourth SARS-CoV-2 mRNA vaccination in anti-CD20-treated multiple sclerosis patients—a longitudinal cohort study
Source: Front Immunol. 2024 Sep 5;15:1432348. doi: 10.3389/fimmu.2024.1432348 (PMC11410621; doi:10.3389/fimmu.2024.1432348)
Supplement: Supplementary file 1 [file Presentation1.pdf]

Supplementary material

Supplementary 1: Quantitative and qualitative assay description (PK and ADA)

ICD 723 Info: (PK)

Quantitative assay to detect Ocrelizumab in human serum. Standard curve is prepared fresh on day of analysis in standard dilution buffer (1% pooled normal human serum/1% normal goat serum in BA011 + 50 ug/mL Goat IgG). Quality controls (QC's) and unknown samples are diluted to assay MRD of 1/100 with sample diluent (1% normal goat serum in BA011 +50ug/mL goat IgG). The calibrators, matrix blank, diluted controls and study samples are transferred to an ELISA plate coated with goat anti-rhuMAb 2H7 polyclonal antibody. During 1hr incubation, ocrelizumab in samples is bound to the immobilized goat anti-rhuMAb 2H7 polyclonal antibody. Unbound materials are removed with wash step. A solution of goat anti human IgG antibody conjugated to HRP is then added and incubated for 1 hr. Any unbound material is removed after final wash step. TMB substrate is added to the plate to develop color. The substrate development is stopped after approximately 10 to 20min by adding 1M Phosphoric acid. Plate is read on a plate reader at 450nm for detection absorbance and at 630nm for reference absorbance.

A nominal Ocre conc range of 156 to 5000ng/mL (neat serum conc) was chosen for sample quantitation. Calibration standards range from 1.56 to 100ng/mL (in well conc.) with 100ng/mL serving as the anchor calibrator. Cal standards are: 1.56, 3.13, 6.25, 12.5, 25, 50, and 100ng/mL (Cal1-7). LLOQ is 1.56ng/mL and ULOQ is 50ng/mL. The Quality controls (QC's) are 250, 1500, and 3500ng/mL (QC1-3). Each QC is frozen in single use aliquots of 50ul for daily use. The MRD for the assay is 1/100. Two-step MRD is performed by adding 20uL of each QC or Sample to 180uL of sample diluent for an initial dilution of 1/10, then adding 50uL of 1/10 diluted QC or samples to 450ul of sample diluent for final MRD of 1/100. Sample dilutions beyond MRD are performed in standard dilution buffer (1% pooled normal human serum/ 1% normal goat serum + 50ug/mL goat IgG). Matrix blanks and calibrators do not receive MRD. The standard dilution buffer is used as the Matrix Blank (MB) on the assayed plate.

ICDIM 172 Info: (ADA)

Qualitative assay to detect antibodies to ocrelizumab in human serum from individuals with MS and uses 2 conjugated reagents to capture antibodies directed against ocrelizumab: biotin conjugated to rhuMAb 2H7 and Digoxigenin (DIG) conjugated to rhuMAb 2H7. The 2 conjugated reagents are co-incubated overnight at room temp with diluted controls and samples. Following overnight incubation, the control (or sample)/biotin/dig solution is transferred to a prewashed streptavidin coated high bind plate and incubated at room temperature. Following wash step, a solution of horseradish peroxidase (HRP) conjugated to chicken anti-digoxin antibody is added for detection to appropriate wells of the washed streptavidin coated plate and incubated at room temperature. After a final wash step, a peroxidase substrate (tetramethylbenzidine) is added to plate for color development and the reaction is stopped by adding 1M phosphoric acid. The plate is read on a plate reader at 450nm (detection) with a 620nm reference filter.

The minimum required dilution (MRD) for the assy is 1/20 in sample diluent and is performed by adding 20uL of each control/sample to 380uL of sample diluent. During screening (tier 1) analysis, all study samples are screened at MRD. Samples with OD responses below screening cut point (mean normalization control (NC) x established screening cut point factor (SCPF), 1.15) are considered negative.

Samples with OD response above screening cut point are considered potentially positive and are analyzed further at Tier 2 confirmatory. Potentially positive samples from screening tier 1 analysis with raw OD responses below SHPC maximum OD response are evaluated at tier 2 confirmatory analysis. Samples are diluted 1/10 with sample diluent and mixed with an equal volume of ocrelizumab confirmatory reagent containing 40ug/mL of ocrelizumab (final drug conc in a sample is 20ug/mL) or sample diluent (drug-uninhibited sample), for a final MRD of 1/20. Samples are confirmed positive for antibodies to ocrelizumab if the percent decrease in signal is at or above 38% using the ocrelizumab confirmatory reagent. Percent decrease is calculated by comparing the raw OD response in the drug inhibited sample to the raw OD response in the drug uninhibited sample. Study samples with raw OD responses at or above SHPC maximum OD response (I.E., SHPC diluted to MRD) in screening (Tier 1) analysis or that confirm positive in confirmatory (tier 2) analysis are titered in Tier 3 analysis until a response below the titer cut point (Mean NC = established titer offset value, 0.0200) is obtained. Following tier 3 analysis, screening samples with raw OD responses at or above the SHPC max OD response are then analyzed in confirmatory analysis at the dilution factor resulting in a raw OD response closest to the OD response of the SHPC MRD. The titer value of the sample is determined by a log titer data reduction program, will only be reported if the confirmatory tier 2 results confirmed positive.

Pooled NHS is used as NC for this assay. NC is analyzed in tier 1 and tier 3 at the MRD (1/20) in sample diluent in 8 wells. NC is also incubated with and without ocrelizumab confirmatory reagent and analyzed in duplicate on each tier 2 plate. Screening Low Positive Control (SLPC) is prepared with anti-2H7 aCDR monoclonal antibody in pooled NHS at a nominal conc of 9ng/mL. SLPC is analyzed in tier 1 analysis at MRD in sample diluent in duplicate on each screening plate. Screening High Positive Control (SHPC) is prepped at nominal conc of 200ng/mL. For tier 1 analysis, SHPC is diluted to MRD with sample diluent and analyzed in duplicate. For tier 3 analysis, SHPC is diluted to MRD with sample diluent and serially diluted ½, seven times with titer diluent. MRD sample and each dilution of the SHPC are analyzed in tier 3 in one well per dilution. Confirmatory Low and High Positive controls (CLPC and CHPC) are prepped at 11.4ng/mL (CLPC) and 200ng/mL (CHPC). The NC, CLPC, and CHPC incubated with and without Ocrelizumab confirmatory reagents are used as confirmatory controls for tier 2 analysis.
